# Thymic cyst: Is attenuation artifactually increased on contrast-enhanced CT?

**DOI:** 10.3389/fonc.2022.984770

**Published:** 2022-10-31

**Authors:** Chao Wang, Jin Mao, Siyu Yang, Huanhuan Xie, Shan Wang, Ling Hu

**Affiliations:** ^1^ Department of Radiology, The Second Affiliated Hospital, Zhejiang University School of Medicine, Hangzhou, Zhejiang, China; ^2^ Department of Ultrasound, Hangzhou Women’s Hospital, Hangzhou, Zhejiang, China

**Keywords:** thymic cysts, CT, pseudoenhancement, artifacts, misdiagnosis

## Abstract

**Background:**

Thymic cysts are often misinterpreted as thymomas or lymph nodes, then leading to unnecessary thymectomy. The purpose of this study was to investigate how the adjacent large vessels artifactually affected attenuation values of thymic cysts on contrast–enhanced CT (CE-CT).

**Methods:**

In this retrospective study, a total of 84 patients were included with pathological diagnosis of thymic cysts and preoperative CE-CT. Quantitative measurements of the size, CT attenuation of thymic cysts and CT attenuation of adjacent large vessels were performed on preoperative CE-CT. According to the absolute change in attenuation of the cysts between contrast-enhanced and nonenhanced CT, the patients were classified into the groups of artifactual hyper-density, unchanged density, and artifactual hypo-density. CT characteristics were compared between the three groups. Furthermore, multivariable logistic regression analysis was performed to determine the independent factors for artifactual hyper-density.

**Results:**

The group of artifactual hyper-density had smaller short diameter of the cysts, higher postcontrast attenuation values and lower nonenhanced attenuation values of the adjacent large vessel. Furthermore, the multivariable logistic analysis showed that artifactual hyper-density of thymic cysts was negatively associated with nonenhanced attenuation of adjacent large vessel, and positively associated with postcontrast attenuation of adjacent large vessel and postcontrast attenuation of cysts.

**Conclusions:**

Most cases with >20 HU nonenhanced CT attenuation in surgically resected cases. Artifactual hyper-density─pseudo-enhancement phenomenon of thymic cysts was more apparent in higher increasing attenuation of adjacent large vessels on CE-CT. A well understanding of this phenomenon can help reduce preoperative misdiagnosis and unnecessary thymectomy.

## Introduction

With the widespread use of computed tomography (CT) in thoracic imaging, detection of incidental mediastinal lesions has become very common. However, CT showed a low diagnostic accuracy in differentiating thymic cysts from thymic tumors (1). Despite advances in CT techniques, the evaluation of the solitary thymic cyst, especially the cyst of non-water attenuation, is still a diagnostic challenge for radiologists (2). A previous study reported there were > 25% unnecessary thymectomy rates (3). Thymic cysts are often misinterpreted as thymomas or lymph nodes because of attenuation values greater than that of water with its “solid” appearance (3, 4). Furthermore, one of the important CT features applied to distinguish thymic cysts from thymomas or lymph nodes is an increase in the attenuation of the mass after intravenous contrast medium administration. Pseudo-enhancement phenomenon of renal cysts on contrast–enhanced CT (CE-CT) has been widely observed and fully acknowledged to be artifactually increased by marked enhancement of peripheral renal parenchyma (5, 6). However, little is known about how the dense contrast medium passing through mediastinal large vessels affects attenuation values of thymic cysts on contrast-enhanced CT. The purpose of this study was to investigate how the adjacent large vessels artifactually affected attenuation values of thymic cysts and its influencing factors on CE-CT.

## Methods

This retrospective study was approved by the ethics commission of the Second Affiliated Hospital of Zhejiang University School of Medicine, and our institutional ethics commission waived the requirement for informed consent. We searched our retrospectively the pathology patient database for consecutive patients who underwent preoperative CE-CT between January 2010 and November 2021, which resulted in a total of 84 patients with pathological diagnosis of thymic cysts and preoperative CE-CT.

Chest CE-CT was performed on inspiration with the standard chest CT protocol. In general, patients were scanned in the supine position from the cranial to caudal direction from the clavicles to the adrenal gland, using multidetector scanners with 16 or 64 detector rows, with 120 kVp and 120–250 mAs (automatic dose modulation). For CE-CT imaging, about 1.2 mL/kg of iodinated contrast material (Iohexol, GE Healthcare, USA; Ultravist, Bayer, Germany; Iopamidol, Bracco Diagnostics, Italy) was injected intravenously at a rate of 3.0 mL/s. A total of 56 patients were performed arterial and venous phase scans, and 28 patients were performed only arterial phase scans. Arterial phase was obtained after 30 seconds of delay after intravenous injection of contrast material, and venous phase was performed after 60 seconds of delay. Then, axial images of 5 mm thickness were reconstructed. Finally, the reconstructed images were transferred to the picture archiving communication system (PACS) workstation.

**Figure 1:**
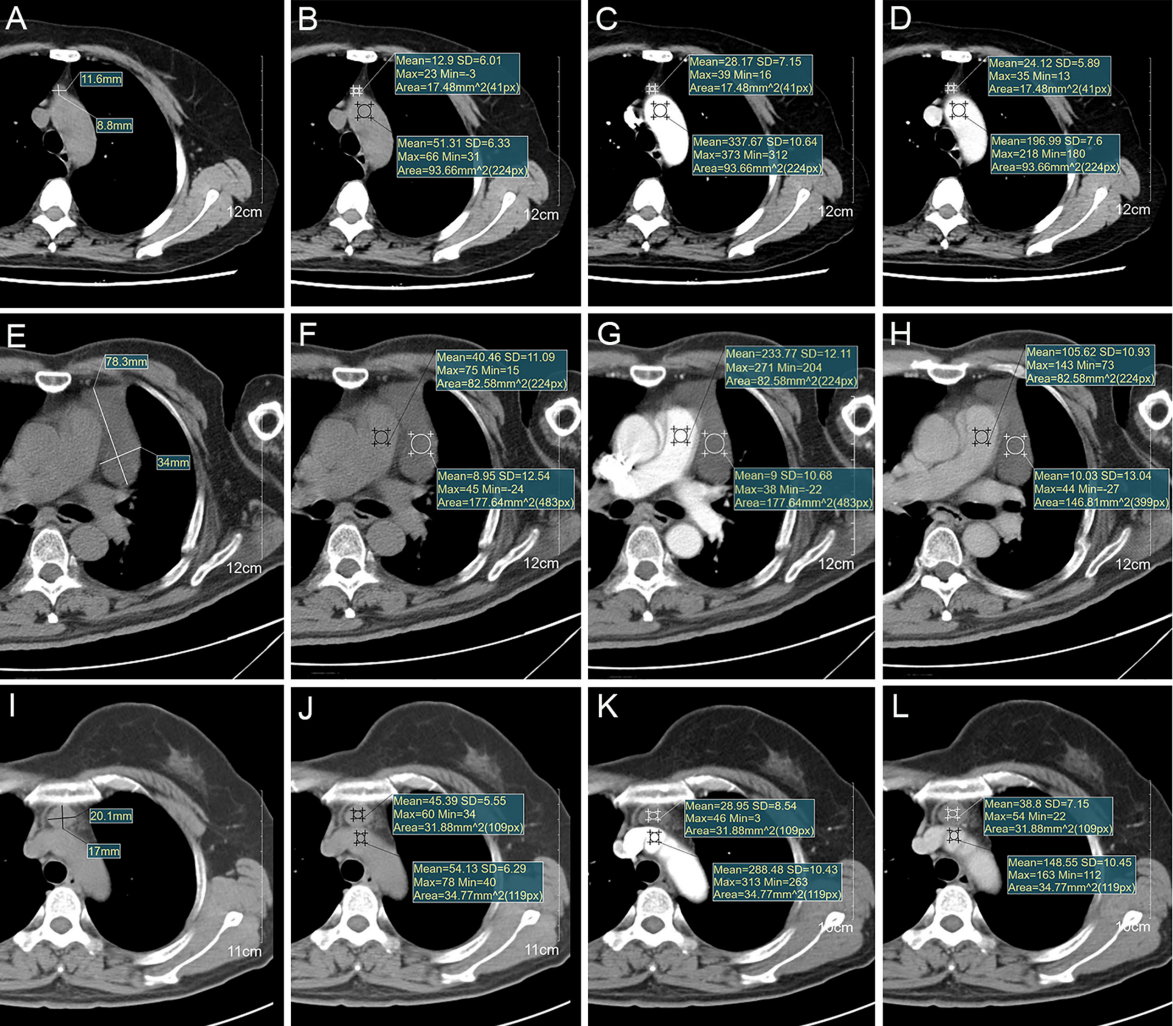
**(A-D)** Images of a thymic cyst with increased postcontrast attenuation values on CE-CT in 62-year-old woman (age in 2018). Imaging shows **(A)** a thymic cyst measuring 11.6×8.8 mm, **(B)** 12.9HU with aortic arch measuring 51.3HU on nonenhanced CT, **(C)** 28.2HU with aortic arch measuring 337.7HU on arterial phase, and **(D)** 24.1HU with aortic arch measuring 197.0HU on venous phase of CE-CT. **(E-H)** Images of a thymic cyst with stable postcontrast attenuation values on CE-CT in 65-year-old man (age in 2017). Imaging shows **(E)** a thymic cyst measuring 78.3×34 mm, **(F)** 9.0HU with main pulmonary artery measuring 40.5HU on nonenhanced CT, **(G)** 9.0HU with main pulmonary artery measuring 233.8HU on arterial phase, and **(H)** 10.0HU with main pulmonary artery measuring 105.6HU on venous phase of CE-CT. **(I-L)** Images of a thymic cyst with decreased postcontrast attenuation values on CE-CT in 53-year-old woman (age in 2018). Imaging shows **(I)** a thymic cyst measuring 20.1×17.0 mm, **(J)** 45.4HU with brachiocephalic trunk measuring 54.1HU on nonenhanced CT, **(K)** 29.0HU with brachiocephalic trunk measuring 288.5HU on arterial phase, and **(L)** 38.8HU with brachiocephalic trunk measuring 148.6HU on venous phase of CE-CT.

Quantitative measurements of the size and CT attenuation of the thymic cysts were performed on the PACS by a board-certified radiologist (SW) with 6 years of experience in chest imaging. This radiologist independently measured the sizes and attenuation of the cysts, and she was blinded to any information about the purpose of the study to reduce a potential bias in measurement. The longest long diameter and the longest short diameter perpendicular to the long diameter of the lesion were obtained at the same largest cross-sectional area (**Figure 1**). CT attenuation values of cysts were measured by placing an oval region of interest (ROI) within the cysts. The ROIs used for the pre- and postcontrast evaluation of a given cyst were obtained at the same cross-sectional location. The mean CT attenuation of each cyst were recorded. The change in attenuation of the cysts were calculated as the absolute difference between the mean contrast-enhanced and nonenhanced attenuation values. Postcontrast CT attenuation values of the adjacent large vessels in both arterial and venous phase were measured by placing an oval ROI within the adjacent large vessels. Then, according to the absolute change in attenuation of the cysts between contrast-enhanced and nonenhanced CT, the patients were classified into three groups, including the groups of > 5 HU (n=27), -5~5 HU (n=38), and <-5 HU (n=19).

Statistical analyses were performed using IBM SPSS Statistics 23 software (IBM Corporation, New York). The normality of the data was tested using Kolmogorov‐Smirnov tests. Normally distributed data were assessed using parametric one‐way analysis of variance (ANOVA) or *t* test. *Post hoc* tests were performed after ANOVA. If the assumption of normality was not met, non-parametric Kruskal‐Wallis H tests or Mann–Whitney U tests were used to assess between-group differences. In addition, chi-square tests were used to evaluate categorical variables. *Post-hoc* analysis with the least significant difference method was performed to further identify where the difference came from. Moreover, Pearson’s correlation analysis was performed to assess correlations between nonenhanced CT attenuation of the cysts and cysts size, and between postcontrast CT attenuation change of thymic cysts and nonenhanced/postcontrast attenuation values of adjacent large vessel. Furthermore, the factors with p < 0.05 were included in the multivariable binary logistic regression analysis to determine the independent factors to differentiate artifactual hyper-density from unchanged- and artifactual hypodensity density. The accuracy odds ratio (OR) and 95% confidence interval (CI) were recorded. All tests were two-tailed and results were considered significant at p<0.05.

## Results

The study group consisted of 84 patients (33 men, 51 women; mean age, 54 ± 11 years; range, 33–82 years). The diagnosis-treatment interval time ranged from 1 to 17 days (mean, 5.2 ± 3.2 days). Nonenhanced CT attenuation of the cysts ranged from 0.6 to 69.2 HU (mean, 33.0 ± 17.5 HU) (**Table 1**). In 62 out of 84 patients (73.8%), the nonenhanced CT attenuation was >20 HU, the threshold usually used to differentiate fluid from soft tissue (**Figure S1**). The thymic cysts ranged from 5.9 to 135.2 mm in long diameter (mean, 31.8 ± 23.4 mm), and from 5.3 to 72.4 mm in short diameter (mean, 20.3 ± 13.2 mm). Nonenhanced CT attenuation of cysts was negatively correlated with the long diameter (r=-0.400, p<0.001) and short diameter (r=-0.396, p<0.001). The change in attenuation of the cysts between contrast-enhanced and nonenhanced CT ranged from -27.4 to 44.3 HU (mean, 2.3 ± 11.2 HU).

**Table 1:** Cyst size, attenuation of thymic cysts and attenuation of the adjacent large vessel.

Parameter	Total No. of Measurement	Value
Cyst size (mm)		
Long diameter	84	31.8 ± 23.4 (24.2) [5.9 to 135.2]*
Short diameter	84	20.3 ± 13.2 (15.9) [5.3 to 72.4]*
CT attenuation of cysts (HU)		
Nonenhanced CT	84	33.0 ± 17.5 (36.2) [0.6 to 69.2]*
Contrast-enhanced CT in arterial phase	84	34.1 ± 19.9 (36.1) [-17.8 to 74.1]*
Contrast-enhanced CT in venous phase	56	37.0 ± 22.9 (38.9) [-2.3 to 97]*
Contrast-enhanced attenuation change in arterial phase	84	1.1 ± 11.3 (0.3) [-27.4 to 39.2]*
Contrast-enhanced attenuation change in venous phase	56	4.2 ± 10.8 (1.8) [-14.0 to 44.3]*
Contrast-enhanced attenuation change in arterial and venous phase†	140	2.3 ± 11.2 (0.5) [-27.4 to 44.3]*
CT attenuation of adjacent large vessel (HU)		
Nonenhanced CT	84	46.8 ± 6.2 (46.9) [19.0 to 57.9]*
Contrast-enhanced CT in arterial phase	84	286.2 ± 58.8 (289.9) [140.5 to 492.3]*
Contrast-enhanced CT in venous phase	56	150.6 ± 24.7 (150.8) [94.3 to 210.3]*

*Numbers are means ± standard deviations, with medians in parentheses and ranges in brackets.

†The absolute difference between the mean contrast-enhanced and nonenhanced attenuation values of cysts in both arterial and venous phase.

**Figure 2:**
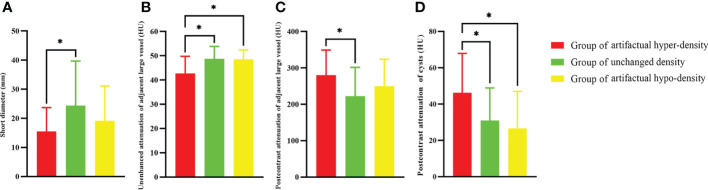
The group difference between the groups of artifactual hyper-density, unchanged density, and artifactual hypo-density according to the CT attenuation change of thymic cysts between contrast-enhanced and nonenhanced CT. **(A)** The group of > 5 HU had smaller short diameter (p=0.008), **(B)** lower nonenhanced attenuation values of adjacent large vessel than the group of -5~5 HU (p < 0.001) and the group of <-5 HU (p=0.001), **(C)** higher postcontrast attenuation values of adjacent large vessel than the group of -5~5 HU (p < 0.001), and **(D)** higher postcontrast attenuation values of cysts than the group of -5~5 HU (p < 0.001) and the group of <-5 HU (p < 0.001). * indicates significant differences.

In addition, according to the change in attenuation of the cysts between contrast-enhanced and nonenhanced CT, the patients were classified into three groups, including the groups of > 5 HU (artifactual hyper-density), -5~5 HU (unchanged density), and <-5 HU (artifactual hypo-density) (**Table 2**). There was significant difference of short diameter among these three groups (p=0.025). Then, *post hoc* tests showed the group of artifactual hyper-density had smaller short diameter than the group of unchanged density (p=0.008) (**Figure 2A**). Furthermore, there were significant differences of nonenhanced attenuation values of adjacent large vessel (p<0.001), postcontrast attenuation values of adjacent large vessel (p<0.001) and postcontrast attenuation values of cysts (p<0.001) among the three groups. Then, *post hoc* tests showed the group of artifactual hyper-density had lower nonenhanced attenuation values of adjacent large vessel than the group of unchanged density (p<0.001) and the group of artifactual hypo-density (p=0.001) (**Figure 2B**); the group of artifactual hyper-density had higher postcontrast attenuation values of adjacent large vessel than the group of unchanged density (p<0.001) (**Figure 2C**); and the group of artifactual hyper-density had higher postcontrast attenuation values of cysts than the group of unchanged density (p<0.001) and the group of artifactual hypo-density (p<0.001) (**Figure 2D**). In addition, the multivariable logistic analysis showed that nonenhanced attenuation of adjacent large vessel (OR: 0.731, 95% CI: 0.611, 0.875), postcontrast attenuation of adjacent large vessel (OR: 1.015, 95% CI: 1.003, 1.027) and postcontrast attenuation of cysts (OR: 1.048, 95% CI: 1.006, 1.092) were identified as independent predictors of artifactual hyper-density (**Table S1**). Correlation analysis showed postcontrast attenuation change of thymic cysts was negatively correlated with nonenhanced attenuation values of adjacent large vessel (r=-0.299, p=0.006), and postcontrast attenuation change of thymic cysts was positively correlated with postcontrast attenuation values of cysts (r=0.488, p<0.001).

**Table 2:** Comparison of clinical characteristics and CT findings between the groups with different absolute difference between the mean contrast-enhanced and nonenhanced attenuation values of thymic cysts.

Parameter	Group of Artifactual Hyper-density	Group of Unchanged density	Group of Artifactual Hypo-density	*P* Value
**Total No. of patients (n)**	27	38	19	
**Age (years)**	55.9 ± 9.1	51.0 ± 10.6	59.0 ± 12.5	0.023
**Sex**				0.025
Men	5	18	10	
Women	22	20	9	
**Diagnosis-treatment interval time**	5.2 ± 2.5	5.3 ± 3.7	4.9 ± 3.0	0.833
**Cyst Size**				
Total No. of cysts (n)	27	38	19	
Long diameter (mm)	23.7 ± 12.6	36.4 ± 24.7	34.3 ± 29.7	0.086
Short diameter (mm)	15.5 ± 8.2	24.3 ± 15.4	19.2 ± 11.9	0.025
**Nonenhanced CT attenuation**				
Total No. of plain scans (n)	27	38	19	
CT attenuation of cysts (HU)	30.7 ± 16.9	32.1 ± 17.6	38.2 ± 17.9	0.322
CT attenuation of adjacent large vessel (HU)	42.8 ± 7.0	48.7 ± 5.1	48.5 ± 3.9	<0.001
**Contrast-enhanced CT attenuation of both arterial and venous phase**				
Total No. of arterial and venous phase scans (n)	47	68	25	
CT attenuation of cysts (HU)	46.2 ± 21.7	30.9 ± 18.0	26.6 ± 20.4	<0.001
CT attenuation of adjacent large vessel (HU)	280.3 ± 68.8	222.5 ± 78.88	249.6 ± 74.2	<0.001

According to the absolute difference in attenuation of the cysts between contrast-enhanced and nonenhanced CT, the patients were classified into the three groups of artifactual hyper-density (> 5 HU), unchanged density (-5~5 HU), and artifactual hypo-density (<-5 HU).

## Discussion

This study provided the clinical, radiological features of surgically resected and histopathologically confirmed thymic cysts. Moreover, this study investigated the influencing factors of artifactually affecting attenuation values of thymic cysts on CE-CT. This study showed most cases (73.8%) with >20 HU nonenhanced CT attenuation, the threshold usually used to differentiate fluid from soft tissue. Nonenhanced CT attenuation of cysts was negatively correlated with cysts size. In addition, the results showed that artifactual hyper-density of thymic cysts was negatively associated with nonenhanced attenuation of adjacent large vessel, and positively associated with postcontrast attenuation of adjacent large vessel and postcontrast attenuation of cysts.

In this study, patients showed a mean age of 54 years with a range of 33 to 82 years, which is similar to the previously reported age range of patients with thymic tumors (7–10). Therefore, age is not a reliable diagnostic factor for differentiating thymic cysts from thymic tumors. This cohort showed female predominant with 51 females and 33 males, which is similar to the previously reported gender predominant (4).

In this study cohort, 73.8% of patients showed nonenhanced CT attenuation was >20 HU, with a mean attenuation of 33 HU range from 0.6 to 69.2 HU. On nonenhanced CT, CT attenuation is much higher than “water/fluid attenuation” which is often used to describe the characteristic appearance of cysts (11). In this study, we found CT attenuation of most thymic cysts were comparable to that of soft tissue lesions rather that water/fluid, which is consistent with the finding of another previous study (4). Among thymic lesions, thymic epithelial tumors and other thymic malignancy is typically considered CT attenuation of soft tissue lesions (>20 HU). As a result, a thymic lesion with a high CT attenuation may be suspicious for solid tumors than cysts by an inexperienced radiologist or thoracic surgeon, then resulting in unnecessary thymectomy.

Both hyper- and hypodense artifacts are common CT artifacts after contrast media injection in chest imaging. In this study, we found that both hyper- and hypodense artifacts affected the attenuation values of the thymic cysts. This study showed that artifactual hyper-density ─ the pseudo-enhancement of thymic cysts was associated with smaller short diameter of the cysts, higher postcontrast attenuation values, and lower nonenhanced attenuation values of the adjacent large vessel. A host of technical factors such as partial volume effect and beam hardening effect due to marked enhancement of adjacent tissue can cause the attenuation change of lesions and result in pseudo-enhancement on CE-CT (5, 12). Maki et al. found renal cyst attenuation increased consistently with increasing background attenuation (12). Similarly, our results firstly confirmed that thymic cysts attenuation increased consistently with increasing attenuation of the adjacent large vessel. Furthermore, our results showed postcontrast attenuation change of thymic cysts was negatively correlated with nonenhanced attenuation values of adjacent large vessel. Additionally, Bae et al. found pseudo-enhancement phenomenon was more apparent in small renal cysts than in large renal cysts (5). Although the multivariable logistic analysis showed small short diameter was not an independent predictor of artifactual hyper-density, ANOVA analysis between groups found the group of artifactual hyper-density had smaller short diameter than the group of unchanged density. The long axis of thymic cysts is usually appressed against adjacent mediastinal large vessels. So, the smaller the diameter of thymic cysts, the closer it is to the adjacent large vessels. Thus, pseudo-enhancement phenomenon of thymic cysts may be more apparent in small short diameter.

As a study limitation, as this study focused on the population of thymic cysts that were histopathologically confirmed after thymectomy, the study population is certainly skewed and represented one end of the spectrum of thymic cysts where the imaging diagnosis suspected to be solid tumors rather than cysts. However, this study was designed to focus on the pathologically confirmed cases, as there has been no specialized study focusing on the CT artifacts findings of histopathologically confirmed thymic cysts.

## Conclusion

This study showed most cases (73.8%) with >20 HU nonenhanced CT attenuation in surgically resected cases, the threshold usually used to differentiate fluid from soft tissue. Nonenhanced CT attenuation of cysts was negatively correlated with cysts size. In addition, artifactual hyper-density─pseudo-enhancement phenomenon of thymic cysts was more apparent in higher increasing attenuation of adjacent large vessels on CE-CT. Pseudo-enhancement phenomenon of thymic cysts is still not well understood. A well understanding of the pseudo-enhancement phenomenon can help reduce preoperative misdiagnosis and reduce unnecessary thymectomy. If pseudo-enhancement phenomenon of thymic cysts is suspected, further MRI or regular CT follow-up should be recommended.

## Data availability statement

The original contributions presented in the study are included in the article/**Supplementary Material**. Further inquiries can be directed to the corresponding authors.

## Ethics statement

The studies involving human participants were reviewed and approved by The ethics committee of the Second Affiliated Hospital of Zhejiang University School of Medicine. The ethics committee waived the requirement of written informed consent for participation.

## Author contributions

CW and LH conceived and designed this paper. CW wrote the draft. JM and SW performed the study and analyzed the data. SY contributed to the literature research. SY and HX revised the draft. All the authors read and approved the final manuscript.

## Funding

This research was supported by Zhejiang Provincial Natural Science Foundation of China under Grant No. LY21H180003.

## Acknowledgments

We would like to thank all colleagues for helping us during the current study and all the volunteers who participated in the study.

## Conflict of interest

The authors declare that the research was conducted in the absence of any commercial or financial relationships that could be construed as a potential conflict of interest.

## Publisher’s note

All claims expressed in this article are solely those of the authors and do not necessarily represent those of their affiliated organizations, or those of the publisher, the editors and the reviewers. Any product that may be evaluated in this article, or claim that may be made by its manufacturer, is not guaranteed or endorsed by the publisher.

## References

[1] Tomiyama N Honda O Tsubamoto M Inoue A Sumikawa H Kuriyama K. Anterior mediastinal tumors: diagnostic accuracy of CT and MRI. Eur J Radiol (2009) 69(2):280–8. doi: 10.1016/j.ejrad.2007.10.002 18023547

[2] Ackman JB Chintanapakdee W Mendoza DP Price MC Lanuti M Shepard JO. Longitudinal CT and MRI characteristics of unilocular thymic cysts. Radiology (2021) 301(2):443–54. doi: 10.1148/radiol.2021203593 34427460

[3] Ackman JB Verzosa S Kovach AE Louissaint A Jr. Lanuti M Wright CD. High rate of unnecessary thymectomy and its cause. can computed tomography distinguish thymoma, lymphoma, thymic hyperplasia, and thymic cysts? Eur J Radiol (2015) 84(3):524–33. doi: 10.1016/j.ejrad.2014.11.042 25575742

[4] Araki T Sholl LM Gerbaudo VH Hatabu H Nishino M. Intrathymic cyst: Clinical and radiological features in surgically resected cases. Clin Radiol (2014) 69(7):732–8. doi: 10.1016/j.crad.2014.03.002 PMC4068340 24824976

[5] Bae KT Heiken JP Siegel CL Bennett HF. Renal cysts: Is attenuation artifactually increased on contrast-enhanced CT images? Radiology (2000) 216(3):792–6. doi: 10.1148/radiology.216.3.r00se14792 10966713

[6] Mileto A Nelson RC Samei E Jaffe TA Paulson EK Barina A. Impact of dual-energy multi-detector row CT with virtual monochromatic imaging on renal cyst pseudoenhancement: *In vitro* and *in vivo* study. Radiology (2014) 272(3):767–76. doi: 10.1148/radiol.14132856 24844472

[7] Jeong YJ Lee KS Kim J Shim YM Han J Kwon OJ. Does CT of thymic epithelial tumors enable us to differentiate histologic subtypes and predict prognosis? AJR Am J Roentgenol (2004) 183(2):283–9. doi: 10.2214/ajr.183.2.1830283 15269013

[8] McErlean A Huang J Zabor EC Moskowitz CS Ginsberg MS. Distinguishing benign thymic lesions from early-stage thymic malignancies on computed tomography. J Thorac Oncol (2013) 8(7):967–73. doi: 10.1097/JTO.0b013e3182904bc2 PMC3978781 23608816

[9] Engels EA Pfeiffer RM. Malignant thymoma in the United States: demographic patterns in incidence and associations with subsequent malignancies. Int J Cancer (2003) 105(4):546–51. doi: 10.1002/ijc.11099 12712448

[10] Sadohara J Fujimoto K Müller NL Kato S Takamori S Ohkuma K. Thymic epithelial tumors: comparison of CT and MR imaging findings of low-risk thymomas, high-risk thymomas, and thymic carcinomas. Eur J Radiol (2006) 60(1):70–9. doi: 10.1016/j.ejrad.2006.05.003 16766154

[11] Jeung MY Gasser B Gangi A Bogorin A Charneau D Wihlm JM. Imaging of cystic masses of the mediastinum. Radiographics (2002) 22:S79–93. doi: 10.1148/radiographics.22.suppl_1.g02oc09s79 12376602

[12] Maki DD Birnbaum BA Chakraborty DP Jacobs JE Carvalho BM Herman GT. Renal cyst pseudoenhancement: Beam-hardening effects on CT numbers. Radiology (1999) 213(2):468–72. doi: 10.1148/radiology.213.2.r99nv33468 10551228

