# Supplementary material for: Thymic cyst: Is attenuation artifactually increased on contrast-enhanced CT?
Source: Front Oncol. 2022 Oct 31;12:984770. doi: 10.3389/fonc.2022.984770 (PMC9671109; doi:10.3389/fonc.2022.984770)
Supplement: Supplementary file 1 [file DataSheet_1.pdf]

Supplementary Materials

Figure S1. A histogram represents the distribution of nonenhanced CT attenuation of thymic cysts in the cohort of 84 patients.

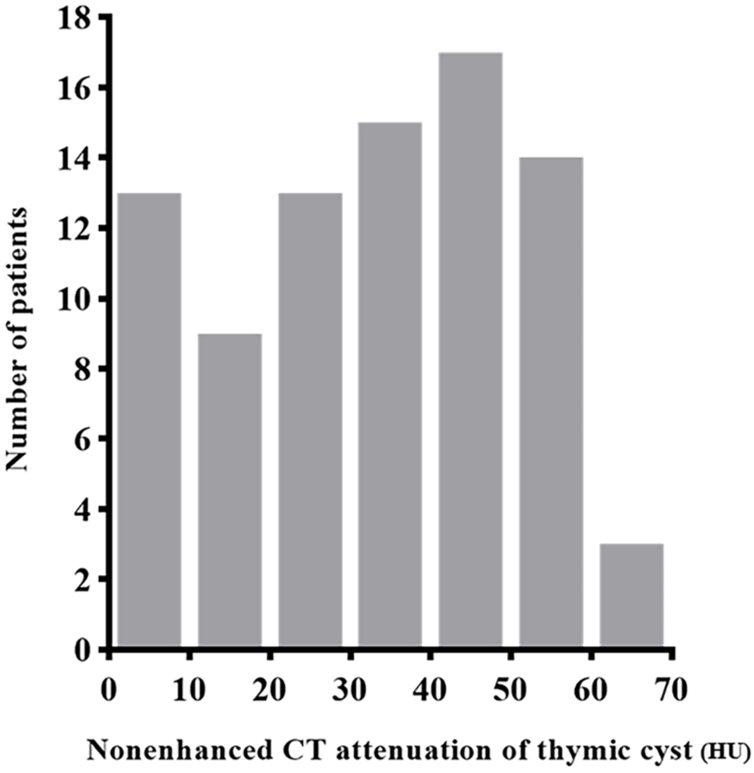

Table S1. Results of multivariable logistic regression analyses

Variables	OR	95% CI	p value
Age	0.999	0.946, 1.055	0.973
Sex	3.876	0.984, 15.269	0.053
Short diameter	0.973	0.906, 1.045	0.452
Nonenhanced CT attenuation of adjacent large vessel	0.731	0.611, 0.875	0.001
Contrast-enhanced CT attenuation of cysts	1.048	1.006, 1.092	0.024
Contrast-enhanced CT attenuation of adjacent large vessel	1.015	1.003, 1.027	0.016

OR, odds ratio; CI, confidence interval
